# Epidemiological Analysis of HIV/AIDS in Kazakhstan During 2018-2020

**DOI:** 10.34172/jrhs.2023.115

**Published:** 2023-06-29

**Authors:** Galiya Bilibayeva, Dinara Ospanova, Anarkhan Nurkerimova, Farida Kussainova, Marat Tukeev, Moldir Shokybaeva, Shynar Tanabayeva, Ildar Fakhradiyev, Timur Saliev

**Affiliations:** ^1^Al-Farabi Kazakh National University, Almaty, Kazakhstan; ^2^Center for Prevention and Control of AIDS, Almaty, Kazakhstan; ^3^S.D. Asfendiyarov Kazakh National Medical University, Almaty, Kazakhstan

**Keywords:** HIV, Prevalence, Infection, Epidemiology, Kazakhstan

## Abstract

**Background:** The human immunodeficiency virus (HIV) is a severe threat to public health everywhere, including the Central Asian region and Kazakhstan. The aim of the study was to conduct an epidemiological analysis of newly diagnosed cases of HIV infection during 2018-2020.

**Study Design:** A case series study.

**Methods:** A descriptive analysis of national data on registered cases of HIV in Kazakhstan was conducted, and demographic information was collected and studied accordingly. The analysis of the influence of age, period, and cohort was performed using the age-period-cohort method.

**Results:** Based on the results, men prevailed (68.5%) among all cases of HIV infection (n=1235). Sexual transmission during heterosexual contact was higher in females (88.9%, *P*=0.005), and the number of new cases as a result of homosexual contact was higher in men (23.0%, *P*=0.087). In addition, the parenteral route of HIV transmission cases prevailed among men (27.5%, *P*=0.001), and intravenous drug administration was more common among males (27.4%, *P*=0.01). Moreover, 68.5% of men and 33.2% of women had a low therapy adherence. In men, the risk of HIV prevalence increased after 32.5 years (deviation [Dv]: 0.134, 95% confidence interval [CI]=0.096 to 0.364). At the age of 37.5 years, there was an increase (Dv: 0.852, 95% CI=0.626 to 1.079) in HIV prevalence. However, no peaks were observed in women.

**Conclusion:** Our findings indicated a rise in the prevalence of HIV infection in Kazakhstan. Men aged 37 and older were identified as the risk category. Eventually, inadequate adherence to treatment was observed in HIV/acquired immunodeficiency syndrome patients.

## Background

 The human immunodeficiency virus (HIV) is a significant threat to global public health.^1^ An estimated 38 million people are living with HIV, 1.7 million new HIV infections and 690.000 acquired immunodeficiency syndrome (AIDS)-related deaths.^2^ Joint United Nations Programme on Acquired Immunity Deficiency Syndrome (UNAIDS) announces a campaign against HIV transmission. It aims to end the HIV epidemic by 2030.^3^ However, the outcomes of fulfilling this objective remain debatable, particularly in developing countries.

 Kazakhstan is one of the largest and fastest-growing post-Soviet nations in Central Asia. Despite recent advancements in the health system due to public policy reforms, bloodborne infectious illnesses, including HIV/AIDS, tuberculosis, and others, continue to be a problem for local healthcare institutions.^4^

 According to UNAIDS data (2019), the number of new HIV infections in Kazakhstan has increased by 39% since 2010. The prevalence rates among the main categories of the population such as people who inject drugs, men who have sex with men, prisoners, and sex workers were 8.5%, 3.2%, 2.7%, and 1.3%, respectively.^5^ However, the study could not reflect the real situation and the actual scale of the epidemic HIV in Kazakhstan due to the lack of accurate data and statistics.^6^ Moreover, it should be taken into account that the country’s cultural specifics (HIV-related stigma and discrimination) might affect the timely diagnostics and treatment of HIV.^7,8^

 It is important to note that information on the prevalence of HIV is not adequately disseminated worldwide. The influence of regional and age differences on the clinical epidemiology of HIV/AIDS has been the subject of a few national studies.^9^ National-scale HIV epidemiology research has not yet been performed in Kazakhstan. The available studies have been limited by localization,^10^ thus they could not provide a broad statistical overview and comprehensive analysis.^11^ Therefore, the interpretation of the results of the study on the territory of the whole country remains difficult.^12^ However, the effective prevention and control of HIV/AIDS continue to be a significant issue due to the lack of reliable national statistics, highlighting the need to analyze incidence trends in relation to the age, period, and cohort (APC) analysis method.^13,14^

 In this regard, this study aimed at conducting an epidemiological analysis of the newly diagnosed cases of HIV infection by gender, age, and treatment adherence in Kazakhstan from 2018 to 2020.

## Methods

###  Ethical issues

 The study was approved by the Local Ethics Committee of the S.D. Asfendiyarov Kazakh National Medical University, Almaty, Republic of Kazakhstan [protocol of the Local Ethics Committee No. 7 (30) dated 30.05.2022].

###  Data collection

 To form the study cohort, we used patient data on registered cases of HIV in 2018-2020. Materials from the electronic registry of patients with HIV, diagnosed from January 1, 2018 to December 31, 2020 throughout the territory of the Republic of Kazakhstan, were provided and approved by the “Republican Centre for the Prevention and Control of AIDS”, Almaty, Kazakhstan.

 The system of epidemiological surveillance for HIV/AIDS in Kazakhstan has been in accordance with the Order of the Minister of Health of Kazakhstan dated October 19, 2020, No. KR DSM-137/2020 on the approval of the rules for taking measures to prevent HIV infection. Diagnosis and treatment of HIV have been based on the clinical protocol for the diagnosis and treatment of the Ministry of Health of the Republic of Kazakhstan dated June 11, 2020 protocol No. 97 “HIV infection in adults”.

 Demographic data such as age and gender of patients were collected and underwent analysis. By age, the patients were divided into 11 groups (20-24, 25-29, 30-34, 35-39, 40-44, 45-49, 50-54, 55-59, 60-64, 65-69, and 70-74 years old).

 According to the place of residence, the detected cases of HIV were studied in the context of 14 regions and 3 cities of administrative significance in Kazakhstan.

 Depending on the routes of transmission, the reported cases of HIV were divided into 4 categories, including sexual transmission through heterosexual contact, sexual transmission during homosexual contact, parenteral transmission, when injecting drugs, and unset path.

 Nonetheless, additional factors were studied, including injecting drug use and alcohol abuse. The clinical stages of HIV infection were determined according to the World Health Organization classification^15^ (Clinical stages 1 to 4).

 Adherence was studied by the frequency of receiving antiretroviral therapy (ART) drugs from the pharmacy. The reasons for low adherence were differentiated into forgetfulness to take medication, feeling better, drugs use, alcohol use, toxic reactions, stigma or lack of privacy for taking ART or personal problems, being too severe due to illness, depression, and other cases. It should be noted that in the territory of Kazakhstan (since 2009), ART treatment, the purchase of antiretroviral drugs for adults and children, has been provided entirely at the expense of public funds. Medical assistance to HIV-infected patients is performed within the guaranteed volume of free medical care.

###  Statistical analysis

 Statistical analysis was performed using SPSS software (version 25.0, IBM SPSS Inc., Chicago, Illinois, USA). The demographic information was obtained from the Statistics Committee of the Ministry of National Economy of Kazakhstan. The data of all HIV cases were used for descriptive analysis of overall trends, geographic location, gender, age, and category. Nonetheless, the analysis was limited to cases for which data were available. Standard data recording procedures were applied to all submitted provincial and territorial datasets to create a national dataset for analysis.

 The epidemiological surveillance data presented in this report have been verified by all provinces and territories to ensure accuracy.

 For the APC analysis, the reported cases of HIV were divided into three years from 2018 to 2020 and twelve age groups (20-24, 25-29, 30-34, 35-39, 40-44, 45-49, 50-54, 55-59, 60-64, 65-69, 70-74, and 75-79 years old).

 The purpose of age, period, and cohort impact analysis is to assess the impact of age, period, and cohort on demographic or incidence rates.^16^ Age effects represent different risks associated with a wide range of age groups. The period effects are changes in vital signs over time that are related to all age groups simultaneously. Cohort effects are associated with changes in indicators in groups of people with the same year of birth (i.e., for consecutive age groups and groups in consecutive periods of time).^17^

 This analysis was performed using the Age-Period-Cohort web tool (Biostatistics Branch, National Cancer Institute, NIH, Bethesda, Maryland).^18^ “Local drifts” were obtained using logarithmic linear regression, and a longitudinal age trend (age trend + periodic trend) and a cross-age trend (age trend - periodic trend) were estimated as well.

 The web tool was used to calculate the relative frequency in any given calendar period (or birth cohort) compared to a control period (or birth cohort) adjusted for age and non-linear cohort (or period) effects. In addition, Wald’s statistical tests were employed to evaluate the studied models.

 The problem with APC identification is that the effects of age, period, and cohort cannot be unambiguously separated due to their linear relationship. However, to solve this problem, the constrained regression (CR) method was applied, introducing restrictions on the coefficients by age, period, and cohort. However, internal scoring and principal components methods were not utilized since they require more data to obtain reliable estimates, and there were limitations in this regard. In addition, some theoretical assumptions were used based on the effects of age, period, and cohort.

###  Age-related impact

 Based on previous research and knowledge of risk factors associated with HIV infection, it is hypothesized that young adults in their 20s and 30s are at higher risk of contracting HIV due to increased sexual activity and other risk factors. This assumption is supported by studies showing a correlation between age and the frequency of HIV infection.^9^

###  Intermittent effects

 It is expected that HIV transmission would decrease once effective prevention and treatment programs are in place. This assumption is based on data on the impact of prevention and treatment programs on reducing HIV incidence in other countries and regions.^19^

###  Cohort effects

 It is assumed that young people born after the introduction of effective HIV prevention programs will have a lower risk of infection. This assumption is based on the observations of how HIV prevention programs can influence knowledge, behaviour, and risk levels from generation to generation.^20,21^

 Considering these theoretical backgrounds, a restricted method (CR) was employed for APC analysis to account for and control for these influences in our model. We introduced constraints on model parameters based on our theoretical assumptions in order to obtain more accurate estimates of the effect of age, period, and cohort on the spread of HIV infection in Kazakhstan.

## Results

 According to population indicators based on the age group, there was a population decline of -11% in the 20-24 age group in 2020 compared to 2018 and a population decline of -7% in the 25-29 age group. The oldest age group of 75-79 years old experienced the greatest population decline (-33%) in 2020 in contrast to 2018. However, a + 27% increase was found in the 70-74-year-old age group. The population had the lowest rate of reduction (-2%) in 2020 as compared to 2018. The total population showed a slight increase up to 1% in 2018 compared to 2020. In the period between 2018 and 2020, n = 1235 HIV cases were registered, of which there were 346 (28%), 396 (32.1%), and 493 (39.9%) cases in 2018, 2019, and 2020, respectively. Depending on gender, the number of men was higher (n = 846, 68.5%) compared to women (n = 389, 31.5%).

 Age and gender characteristics of registered HIV cases in the context of 2018-2020 in Kazakhstan are presented in Table 1. In 2018, 231 and 115 cases of HIV were diagnosed among men and women, respectively. For the same period, HIV cases among men prevailed in the age categories 30-34 years old, accounting for 27 (17.7%), 35-39 (18.6%), and 40-44 (17.3%) years old. Among women in 2018, the diagnosis of HIV was determined more often in the age categories of 30-34 and 35-39 years (n = 23, 20.0%) and n = 29, 25.2%), respectively. In 2019, in the age category of 35-39 years, HIV was detected among 57 men (20.9%) and 26 women (21.1%). Further, in the age group of 30-34 years, cases of HIV registration prevailed among 42 men (15.4%) and 20 women (16.3%). In 2020, the highest incidence of HIV registration among men and women was determined in the age group of 40-44 years, accounting for 65 (19.0%) and 30 (20.0%) cases, respectively. In the age groups of 30-34 and 35-39 years, cases of HIV infection were registered among men in almost 60 cases, while the number of cases among women was 25.

**Table 1:** Age and gender characteristics of registered HIV cases during 2018-2020

**Age (y)**	**2018**		**2019**		**2020**		**Total**	
	**М**	**F**	**М**	**F**	**М**	**F**	**М**	**F**
20-24	7	4	18	9	44	7	69	20
25-29	27	11	44	17	50	13	121	41
30-34	41	23	42	20	61	25	144	68
35-39	43	29	57	26	63	26	163	81
40-44	40	13	40	14	65	30	145	57
45-49	30	16	35	7	28	20	93	43
50-54	15	7	16	11	13	12	44	30
55-59	10	7	10	11	7	12	27	30
60-64	9	2	6	5	6	4	21	11
65-69	5	1	3	3	1	2	9	6
70-74	4	2	0	0	2	0	6	2
75-79	0	0	2	0	2	0	4	0
Total	231	115	273	123	342	151	846	389

*Note.* HIV: Human immunodeficiency virus; M: Male; F: Female.

Table 2 provides types of HIV transmission depending on gender in the context of 2018-2020 on the territory of Kazakhstan. In 2018, the parenteral route of transmission through injecting drugs predominated in 66 (28.6%) of registered cases of HIV in men, whereas the sexual route through heterosexual contacts predominated in 120 women (51.9%). The majority of cases (n = 103, 89.6%) of sexual transmission among women were through heterosexual connections; there were no cases of sexual transmission through homosexual contacts, although there were 44 (19.1%) cases of sexual transmission through gay contacts among males. For the same period of time, among women and men with HIV, sexual transmission through heterosexual contacts occurred in 115 (93.5%) and 129 (47.2%) cases, respectively. In 2019, the sexual route of transmission among men through gay relationships was documented in 68 (24.9%) cases, while the parenteral route of HIV transmission was recognized in 75 (27.5%) cases. The sexual route of HIV infection through heterosexual interactions predominated in both genders in 2020, and women (84.8%) had this mode of transmission more than twice as often as men (48.2%). However, males were more likely than females to take drugs by injecting them [n = 92 (26.9%) and n = 21 (13.9%)], respectively. Therefore, from 2018 to 2020, women (88.9%) were more likely than men (48.9%) to transmit sexually through heterosexual interactions (*P* = 0.005). Only one (0.3%) case among women and 195 (23.0%) male cases who had gay relations used the sexual route of transmission, with no statistically significant difference (*P* = 0.087). In 233 (27.5%) cases, more men than women (n = 41, 10.5%) contracted HIV via the parenteral route. Such a difference was statistically significant (*P* = 0.001).

**Table 2:** Types of HIV transmission depending on gender in the context of 2018-2020 on the territory of Kazakhstan

**Transmission types**	**2018**		**2019**		**2020**		**Total**		***P*** ** value**
	**М**	**F**	**М**	**F**	**М**	**F**	**М**	**F**	
	**n=231**	**n=115 **	**n=273**	**n=123 **	**n=342**	**n=151 **	**n=846**	**n=389 **	
Sex with heterosexual contacts	120	103	129	115	165	128	414	346	0.005
Sex with homosexual contacts	44	0	68	0	83	1	195	1	0.087
Parenteral transmission when injecting drugs	66	12	75	8	92	21	233	41	0.001
Unknown type	1	0	1	0	2	1	4	1	0.072

*Note.* HIV:Human immunodeficiency virus; M: Male; F: Female.

 Indicators showing the frequency of injecting drug use and alcohol use among HIV diagnoses, broken down by gender, during 2018-2020 are summarized in Table 3. Injection drug usage rates in 2018 represented that there were more injecting drug users in a group of 66 (28.6%) men with HIV compared to the group including 12 (10.4%) women. The rates of alcohol abuse in 2018 among men and women were only observed in 4 (1.7%) and 3 (2.6%) cases, respectively. In 2019, 75 men (27.5%) used drugs more frequently than 8 women (6.5%), demonstrating a difference of more than four times. The prevalence of alcohol abuse among people with HIV reported cases in 2019 across men and women was found to be nearly equal [n = 6 (2.2%) and n = 3 (2.4%), respectively]. In 2020, regarding injecting drug use among people with registered cases of HIV, in women [n = 21 (13.9%)], it was observed almost half as much as in men [n = 91, (26.6%)]. Furthermore, alcohol abuse was more often identified in 30 (8.8%) men in contrast to 5 (3.3%) women. Thus, on average, injecting drug use was more common among males [n = 232 (27.4%)] compared to females [n = 41 (10.5%)] in 2018-2020, with a statistically significant difference (*P* = 0.005). Additionally, alcohol abuse was more often detected among 40 (4.7%)] men and only in 11 (2.8%) women; however, no statistically significant differences were found in this respect (*P* = 0.095).

**Table 3:** Indicators of the frequency of injecting drug/alcohol use in diagnosed cases of HIV depending on gender in the context of 2018-2020 on the territory of Kazakhstan

**Indicators**	**2018**		**2019**		**2020**		**Total**		***P*** **-value**
	**М**	**F**	**М**	**F**	**М**	**F**	**М**	**F**	
	**n=231**	**n=115 **	**n=273**	**n=123 **	**n=342**	**n=151 **	**n=846**	**n=389 **	
Injection drug use									
Yes	66	12	75	8	91	21	232	41	0.005
No	165	103	198	115	251	130	614	348	0.231
Alcohol abuse									
Yes	4	3	6	3	30	5	40	11	0.095
No	227	112	267	120	312	146	806	378	0.432

*Note.* HIV:Human immunodeficiency virus; M: Male; F: Female.

Table 4 lists the clinical phases of diagnosed HIV patients by gender in Kazakhstan from 2018 to 2020. In the vast majority of cases, clinical stage 1 HIV was identified in 142 (61.5%) men and 80 (69.5%) women in 2018. HIV detection rates for men and women at stages 2 and 3 were 13.4% and 11.3%, as well as 19.5% and 15.6%, respectively. In 2019, the first clinical stage of HIV was frequently observed for both genders [163 (59.7%) men and 83 (67.5%) women]. Moreover, stage 3 HIV was detected = 51 men (18.7%) as opposed to 18 women (14.6%). In 2020, there were 103 (68.2%) more stage 1 HIV diagnoses in women than in men (n = 201, 58.8%). Contrarily, men were more likely to experience the 2nd clinical stage of HIV infection [n = 71 (20.8%)] compared to women [n = 20 (13.2%)]. Only 19 (5.5%) cases of men reached the 4th clinical stage of HIV in 2020 compared to 11 (7.3%) cases of women. Consequently, on average, 266 (68.4%) cases of stage 1 HIV were found in females in comparison to 506 (59.8%) cases in males between 2018 and 2020 (*P* = 0.627). Only 54 (13.9%) women and 150 (17.7%) men had the 2 clinical stage of HIV. However, there was no statistically significant difference between genders (*P* = 0.082). Clinical stage 3 HIV was identified in 147 (17.4%) men and 53 (13.6%) women, respectively (*P* = 0.175). Stage 4 HIV also predominated in men [n = 43 (5.1%)] compared to women [n = 16 (4.1%)], but there was no statistically significant difference between genders (*P* = 0.238).

**Table 4:** Clinical stages of diagnosed HIV cases by gender in the context of 2018-2020 on the territory of Kazakhstan

**Stages**	**2018**		**2019**		**2020**		**Total**		***P*** ** value**
	**М**	**F**	**М**	**F**	**М**	**F**	**М**	**F**	
	**n=231**	**n=115**	**n=273**	**n=123**	**n=342**	**n=151**	**n=846**	**n=389**	
I	142	80	163	83	201	103	506	266	0.627
II	31	13	48	21	71	20	150	54	0.082
III	45	18	51	18	51	17	147	53	0.175
IV	13	4	11	1	19	11	43	16	0.238

*Note.* HIV:Human immunodeficiency virus; M: Male; F: Female.

 Overall, 389 (33.2%) women and 846 (68.5%) men exhibited low adherence in the context of 2018.10.10. Table 5 lists the causes of low HIV adherence by gender from 2018 to 2020 in Kazakhstan. In 2018, forgetfulness to take prescriptions was identified as one of the factors that caused low adherence in 7 men (20.6%), and 5 (14.7%) cases related this with a harmful reaction to drugs. Conversely, among women in 2018, the cause of low adherence was linked to stigma, a lack of privacy when taking ART, or personal issues [n = 6 (33.3%)]. In 2019, in both men [12 (31.6%)] and women [n = 9 (45.0%)], forgetfulness was the reason for low adherence. The association of low adherence with drug use was higher in men [n = 6 (15.8%)] compared to women [n = 2 (1.6%)]. Moreover, other reasons for low adherence were identified among 8 men (21.0%) and 4 women (20.0%) patients. As in the previous two years, in 2020, forgetfulness prevailed as the predominant cause of low adherence in men and women [n = 15 (32.6%) and n = 8 (27.6%), respectively]. In 3 cases, including both genders, a toxic reaction was found to be the root cause of low adherence. In 2020, men [n = 7 (15.2%)] were more likely to experience stigma or a loss of privacy related to getting ART or personal issues than women [n = 4 (13.8%)].

**Table 5:** Reasons for Low HIV adherence by gender on the territory of Kazakhstan during 2018-2020

**Reasons of low adherence**	**2018**		**2019**		**2020**		**Total**	
	**M** **n=231**	**F** **n=115 **	**M** **n=273**	**F** **n=123 **	**M** **n=342**	**F** **n=151 **	**M** **n=846**	**F** **n=389 **
Forgetting to take	7	4	12	9	15	8	34	21
Feeling better	1	0	0	1	0	0	1	1
Drug use	3	1	6	2	3	1	12	4
Alcohol consumption	2	-	6	-	3	-	11	-
Toxic reactions	5	1	2	1	3	3	10	5
Stigma or lack of privacy for ART or personal problems	4	6	3	2	7	4	14	12
Too sick	1	0	0	1	1	4	2	5
Depression	2	1	1	1	1	0	4	2
Other	9	5	8	4	13	9	30	18
Total	34	18	38	20	46	29	118	67

*Note.* HIV:Human immunodeficiency virus; M: Male; F: Female; ART: Antiretroviral therapy.

**Figure 1:**
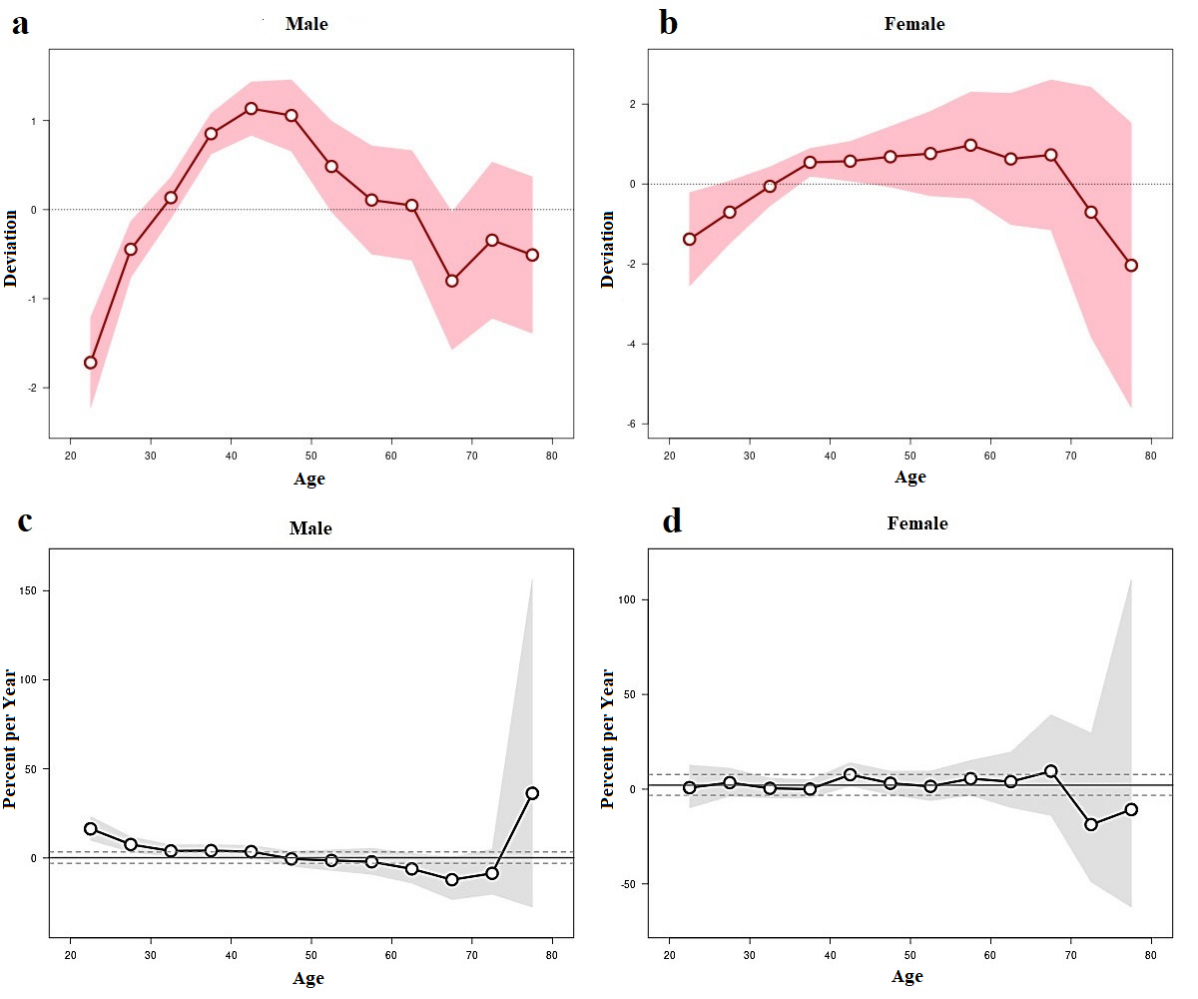
Age-period-cohort analysis: (a) Longitudinal age curves of the level of HIV infection on male gender, (b) Longitudinal age curves of the level of HIV infection on female gender, (c) Local values of HIV drift depending on male gender, and (d) Local values of HIV drift depending on female gender. Note. HIV: Human immunodeficiency virus.

Figure 1(a, b) illustrates the longitudinal age curves of the prevalence of HIV infection (1/1000) and 95% confidence intervals by gender according to a data analysis from the Association for Psychological Science research. The results revealed that after 32.5 years, men are at a higher risk of contracting HIV (Dv: 0.134, 95% CI = -0.096 to 0.364). Additionally, a peak in HIV prevalence was recorded in men at the age of 42.5 years old (Dv: 1.135, 95% CI = 0.836 to 1.433). Men’s HIV prevalence decreased steadily until age 52.5 (Dv: 0.484, 95% CI = -0.023 to 0.992) and then rapidly decreased at age 62.5 (Dv: 0.047, 95% CI = 0.567 to 0.662), finally reaching a minimum at age 67.5 (Dv: -0.8, 95% CI = -1.571 to -0.029), the details of which are displayed in Figure 1a. HIV prevalence increased more slowly in women than in men, and an increase in cases was observed at age 37.5 (Dv: 0.541, 95% CI = 0.196 to 0.887). In addition, until the age of 57.5, there was a consistent upward trend in its prevalence for women (Dv: 0.972, 95% CI = -0.355 to 2.299). Next, there was a minor increase in HIV infections in women at age 67.5 (Dv: 0.729, 95% CI = -1.146 to 2.604), followed by a slight drop in prevalence rates at age 62.5 (Dv: 0.627, 95% CI = -1.013 to 2.267). Further, there was a significant decline in the prevalence of HIV infection in women starting at the age of 72.5 (Dv: -0.705, 95% CI = -3.828 to 2.418), the results of which are depicted in Figure 1 b.

Figure 1(c, d) displays the annual percentage change (%) in HIV prevalence by age group and gender. The findings demonstrated that for both men and women, local drift values varied significantly by the age group. At age 22.5 in men, local drift tended to be positive with a percentage rate of 16.359 per year. The yearly percentage change among men was -0.505% per year at age 47.5, indicating a downward trend. At the age of 72.5 years, the minimum value in percentage per year for the group of men was -8.603. Additionally, a rise in the rates of 36.284% per year was found in men aged 77.5, representing a positive trend in the yearly percentage change value (Figure 1c). The annual percentage change (%) in HIV rates for women was typically positive, with periods of growth of 3.452%, 7.637%, and 9.496% at ages 27.5 and 47.5, respectively.

 At age 72.5, there was a maximum negative percentage change in HIV cases, which was -18.675% per year. At age 77.5, there was a minor uptick to -10.75% per year. As a result, it should be highlighted that neither the figures for men nor those for women over the age of 70 accurately depict the influence of aging on HIV prevalence. However, using specific Wald test results, we found statistically significant effects of cohorts (All Cohort RR = 1, χ^2^ - 53.84, Df-13, *P* = 0.001) and local drift (All Local Drifts = Net Drift, X2 -37, 37, Df -12, *P* = 0.0002) in the male population; however, the effect of periods (All Period RR = 1, χ^2^ - 0.60, Df-2, *P* = 0.738) did not have a statistically significant difference. In the female population, there was only a statistically significant effect of cohorts (All Cohort R 1, χ^2^ -11.16, Df-13, *P* = 0.049), while the effect of periods (All Period RR = 1, χ^2^ - 0, 86, Df-2, *P* = 0.640) and local drift (All Local Drifts = Net Drift, χ^2^ -7.71, Df -12, *P* = 0.806) were not statistically significant (Figure 1d).

## Discussion

 To the best of our knowledge, this is the first epidemiological analysis of HIV prevalence in Kazakhstan performed between 2018 and 2020. It includes research on how age, time, and cohort do affect HIV (APC) prevalence.

 In the context of HIV infection, biological changes such as hormonal fluctuations and changes in the immune system can influence susceptibility to infection with age.^22^ In turn, the social processes of aging, including changing partners, social networks, and behavioural risks, can also influence the risk of acquiring HIV at different ages.^23^ In addition, factors such as the availability and quality of health care, HIV prevention campaigns, and changes in legislation can affect all age groups in the same way and change HIV incidence rates.^24^ In this case, the period effect may be the result of these common factors affecting different age groups in a particular time period.

 In fact, age is one of the most important demographic factors affecting HIV morbidity and mortality.^25,26^ According to some published data, the effect of age on the incidence of AIDS in men was higher than in women.^27^

 In our study, more males than women had HIV diagnoses during 2018-2020. In fact, women are a frequently ignored vulnerable population. Given that women make up more than half (55%) of all HIV/AIDS patients worldwide, they are frequently disproportionately affected by the disease.^28^ Some researchers hypothesized that it might be linked to the higher number of women who regularly visit hospitals (for check-ups) compared to men. Another factor could be associated with the assumption that men are more likely to contract HIV. Males who have intercourse with other men, for example, may be reluctant to visit healthcare facilities out of fear of stigma.^29^

 Our findings showed a substantial age peak in men for sexually transmitted HIV patients between the ages of 35 and 39, pointing to sexual interaction as the primary cause of HIV infection. According to a Chinese study, men and women had significantly different rates of HIV prevalence, occurrence, and death.^30^ According to this study, women indicated the same shape in the curves of the three indicators over time, and men had extremely higher amplitude effects. This is particularly evident in the incidence rate, which peaked in 2005 at 7.71/100 000 males versus 2.86/100,000 females, and the mortality rate, which rose to 3.63/100 000 males versus 1.25/100 000 females in 2017.

 In some countries, the growing proportion of newly confirmed patients attributed to sexual transmission increased from 11% in 2005 to 96% in 2017. Heterosexual transmission increased from 11% to 70%, while homosexual transmission increased from almost 0% to 26%.^31^

 The results demonstrated that sexual transmission through heterosexual contact was often in the female population (2018-2020, *P* = 0.005). The sexual route of transmission during homosexual contact was more often determined among men (*P* = 0.087). The parenteral route of HIV transmission prevailed among men compared to women (*P* = 0.001). The risk of homosexuals in the manifestation of HIV infection is a continuing social problem throughout the world.^32^ Due to the high prevalence of HIV in some countries, homosexual intercourse is socially stigmatized, and epidemiological approaches to this group are difficult.^33^ However, due to the cultural and behavioural characteristics of the inhabitants of the country, as in studies conducted earlier in other countries, the transmission of HIV infection through homosexual contact was not prevailing in comparison with heterosexual sexual intercourse, which caused HIV infection.^34^

 Males were more likely than females to use intravenous drug intake over the 2018-2020 study period (*P* = 0.005). Men were also found to abuse alcohol more frequently (*P* = 0.05). Our findings are in agreement with those of a previous study performed in Taiwan.^35^ Risks related to high-risk sexual behaviour were shown to be more significant in females than the risks associated with injecting drug use. In 12 studies conducted in Kazakhstan, the prevalence of HIV ranged from 0.06% to 30.1% (median 12.0%) according to a previously published review.^36^ In Kazakhstan, the proportion among women injecting narcotics was the highest (30.1%). According to the findings of sizable cohort research performed in Korea, sexual contact was the primary route of HIV transmission in 94% of patients.^37^

 There was no statistically significant difference in the clinical stage of HIV depending on gender.

 According to APC research, men’s HIV prevalence increased significantly around the age of 37.5. The prevalence of HIV in men peaked around age 42.5. No peaks were observed despite a rise in HIV prevalence in women at the age of 37.5.

 Many studies have previously revealed that HIV patients’ lower socioeconomic position is typically related to poor adherence to treatment.^38^ Given that the government in Kazakhstan fully covers the cost of HIV treatment, factors contributing to low adherence may be attributed to patients’ personal circumstances such as forgetfulness.

 This study has some limitations. We employed an APC analysis over three years in the absence of other long-term data. The APC analysis method has been usually employed to study changes over a long period of time. Thus, there is a certain risk of distorting the results if this method is applied to an insufficiently long period of time. Therefore, it is important to continue monitoring and collecting data over a longer period of time in order to more accurately determine trends in the spread of HIV infection in the region.

**Highlights**

- To the best of our knowledge, this is the first epidemiological analysis of HIV prevalence performed in Kazakhstan between 2018 and 2020.
- The study findings indicated a rise in the prevalence of HIV infection in Kazakhstan.
- Men aged 37 and older were identified as the risk category. Inadequate adherence to the treatment was noted in HIV/AIDS patients.
- The findings revealed that tactics for combating HIV infection need to be improved, and preventive measures require optimization.

## Conclusion

 The findings revealed a steadily rising frequency of HIV infection in Kazakhstan. Men aged 37 and older were shown to be the main risk category. In addition, low adherence to the treatment was found in HIV/AIDS patients. The results highlight the need for the development of an efficient strategy to address the HIV infection issue.

## Acknowledgments

 The authors express their gratitude for the administrative and technical support provided by the S.D. Asfendiyarov Kazakh National Medical University.

## Authors’ Contribution

**Conceptualization:** Galiya Bilibayeva, Dinara Ospanova.

**Data curation:** Galiya Bilibayeva, Anarkhan Nurkerimova.

**Formal analysis:** Dinara Ospanova, Farida Kussainova.

**Investigation:** Galiya Bilibayeva, Marat Tukeev, and Moldir Shokybaeva.

**Methodology:** Anarkhan Nurkerimova, Ildar Fakhradiyev, Timur Saliev.

**Project administration:** Dinara Ospanova.

**Supervision**: Galiya Bilibayeva.

**Visualisation:** Moldir Shokybaeva, Marat Tukeev.

**Writing–original draft:** Galiya Bilibayeva, Shynar Tanabayeva, Ildar Fakhradiyev, Timur Saliev.

**Writing–review & editing:** Galiya Bilibayeva, Shynar Tanabayeva, Ildar Fakhradiyev, Timur Saliev.

## Competing Interests

 The authors declare no conflict of interests.

## Funding

 This research received no external funding.
